# Evaluating Health Research Capacity Building: An Evidence-Based Tool

**DOI:** 10.1371/journal.pmed.0030299

**Published:** 2006-07-18

**Authors:** Imelda Bates, Alex Yaw Osei Akoto, Daniel Ansong, Patrick Karikari, George Bedu-Addo, Julia Critchley, Tsiri Agbenyega, Anthony Nsiah-Asare

## Abstract

Bates and colleagues describe the development of a tool to assess capacity-building programs in health research, which they used in Kumasi, Ghana.

An increasingly important goal of governments and external agencies in developing countries is the need for “capacity building” in health research. Although a poorly defined and understood concept, capacity building would essentially enable de novo health research programmes to be facilitated and existing programmes to be strengthened (see [1] and page 14 in [2]). For health research, the goal of building capacity is thus to improve the ability to conduct research, to use results effectively, and to promote demand for research (see page 14 in [2]). Prioritising the need for the international community to make a “quantum leap in capacity building”, as suggested in 1998 by the Director General of the World Health Organization (WHO), would improve health and reduce poverty in developing countries [3].

To achieve this goal, there is an urgent need for an evidence-based tool for determining whether the required infrastructure is present in a given setting, as well as for underpinning the design and evaluation of capacity-building programmes in health research. Here, we describe the development and use of such a tool through analysis of published models and effective capacity-building principles, together with structured reflection and action (see page 9 in [2]) by stakeholders at the Komfo Anokye Teaching Hospital (KATH) in Kumasi, Ghana.

## Challenges Faced in Building and Supporting Research Capacity at KATH

KATH benefits from a new management team that is committed to developing the hospital and medical school into a regional centre of excellence for research, teaching, and clinical care. Although local clinicians had previously been involved in multinational research projects, these projects had largely been generated by external agencies. Local staff lacked experience in the conception and design of projects, and the hospital lacked local role models and tutors for generating de novo research. Consultant posts at KATH remained vacant because senior clinical trainees had difficulty in completing the prerequisite research component of their exit examinations for the West African Colleges. Tellingly, when asked why they had not completed their specialist exams, the most common reason given by health professionals in KATH was apprehension of beginning their own research programmes.

KATH management needed a tool that they could use to ensure that all necessary resources were in place to support local research. Unfortunately, the literature specifically describing the building of health research capacity is scarce and tends to emphasise microlevel activities, such as the choice of research trainees (e.g., Nchinda TC [3]), without considering how these activities can be integrated into the wider research system. Moreover, much of the available information on building research capacity is based on retrospective reports of external consultants, and the perspective of implementing capacity building in a developing country is almost never represented [4]. Our aim therefore was to develop an evidence-based tool that could be used to guide the design, implementation, and evaluation of capacity building in health research programmes.

## Developing an Appropriate Evaluation Tool

**Figure 1:**
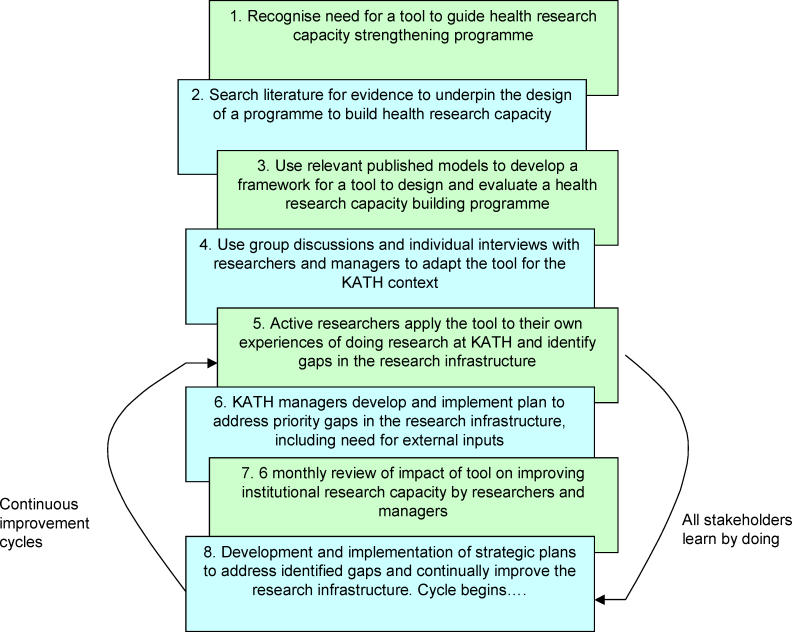
Outline of Programme to Develop and Test Tool for Evaluating Research Capacity at KATH

We used a three-stage approach: (1) searching the literature for existing tools and models; (2) analysing best-practice examples to guide the overall framework; and (3) adapting the framework into an operational tool that met the specific needs of KATH. By using translational research principles to analyse our findings, we systematically extracted and extrapolated stakeholders' evidential and experiential stories (see page 9 [2], and used this information to inform the overall design of our programme. Figure 1 outlines the stages of development and testing of the tool.

### Literature search

We searched the following electronic databases: MEDLINE, Ingenta, and Science Direct using keywords, such as “capacity building”, “capacity development”, “developing countries,” and “Africa”. We retrieved the full text of any relevant papers, including articles cited in the reference lists of these papers. Because there is limited information about health research capacity building in peer-reviewed literature, we also consulted books, Web sites of organisations working on health and research capacity building (e.g., Web sites of WHO, United Nations agencies, the European Community, and the International Development Research Centre), and references provided by colleagues. This evidence was used to derive a definition of health research capacity building, to identify existing capacity-building models, and to synthesise best-practice examples to derive key principles. Dataset S1 gives a detailed overview of our literature review on capacity building.

We found that many different definitions have been applied to capacity building according to the particular level—“micro”, “meso”, or “macro” (focused on in [5])—but that one of the most widely used definitions is “an ability of individuals, organisations or systems to perform appropriate functions effectively, efficiently and sustainably” [6].

By combining the definition for generic “capacity building” with published evidence and our practical experiences of developing a planning and evaluation tool, we have defined building capacity for health research as “an ability of individuals, organisations, or systems to perform and utilise health research effectively, efficiently, and sustainably.”

### Using published best-practice examples to design the evaluation programme

No tools exist that are specific for evaluating health research capacity-building programmes. However, the literature review was helpful for identifying ineffective capacity-building strategies, such as “bolting-on” capacity building to research projects initiated by a specific donor in developing countries [7]. It was also useful for highlighting generic principles underlying successful capacity building.

We grouped the generic principles that consistently emerged from the literature as best practices into themes that emphasised the importance of three concepts. The first theme was a “phased approach”; this requires the sequential involvement of all stakeholders in assessing capacity gaps, developing strategies to fill these gaps, and evaluating outcomes [6]. The second theme was “strengthening of existing processes”; this is an iterative and flexible process that focuses on enhancing local ability to solve problems, define and achieve development needs, and then incrementally incorporate expanding circles of individuals, institutions, and systems [8]. The third theme was “partnerships”; for effective or sustained capacity building, the various partners involved must have similar concepts [5] and share responsibilities and obligations, with local partners taking ownership and leadership [6,9]. Thus, the role of external expertise is to facilitate the development of local skills through learning by experience, rather than acting as a “donor” who retains control of funds and expertise over a poorer “beneficiary” partner.

### Developing and adapting the evaluation tool

An illuminating finding of the literature search was that there was no model that had been specifically designed with health research capacity building in mind. Indeed, the most useful model was one that had been developed for institutionalising quality assurance (QA) [10] because it focused on defining, measuring, and improving quality. This mirrors the processes required for capacity building in health research: defining the institutional systems needed to support research, enumerating existing and missing resources, and improving research support by addressing identified gaps. The QA institutionalisation framework represented a synthesis of over ten years' experience in developing countries, and was derived from a combination of organisational development and QA literature. The framework described organisations as passing through four phases when they implement innovation: awareness, experiential, expansion, and consolidation (Table 1).

**Table 1:**
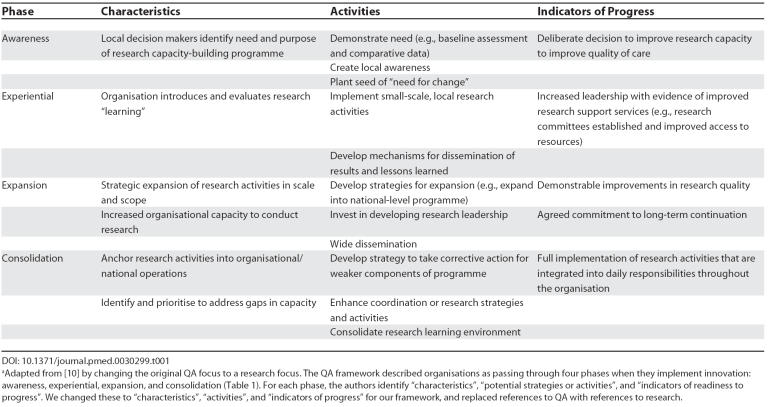
Framework for Designing and Evaluating a Health Research Capacity-Building Programme

In the course of adapting our framework into a tool that was relevant to KATH, we were also influenced by a published framework for dissemination and implementation of evidence-based medicine (EBM) [11]. This prompted us to change the name of the “experiential” phase to “implementation”, as this was more appropriate to a research programme.

To meet the specific needs of KATH, local research stakeholders participated in adapting the tool. These stakeholders comprised ten KATH health professionals (nine clinicians and one physiotherapist), and senior hospital managers—including the chief executive, medical director, and heads of departments. Individual and group discussions took place during a workshop for the health professionals. Stakeholders considered each phase in the framework (Table 2), and suggested characteristics, activities, and indicators of progress in building research capacity that met the needs of KATH and that could be feasibly measured or shown. The stakeholders' suggestions were incorporated into the framework to create an operational tool that could be used to identify gaps in the research infrastructural support at KATH (Table 2). This ensured that a holistic approach was taken to developing the research capacity in the hospital, rather than a fragmented, unfocused approach.

**Table 2:**
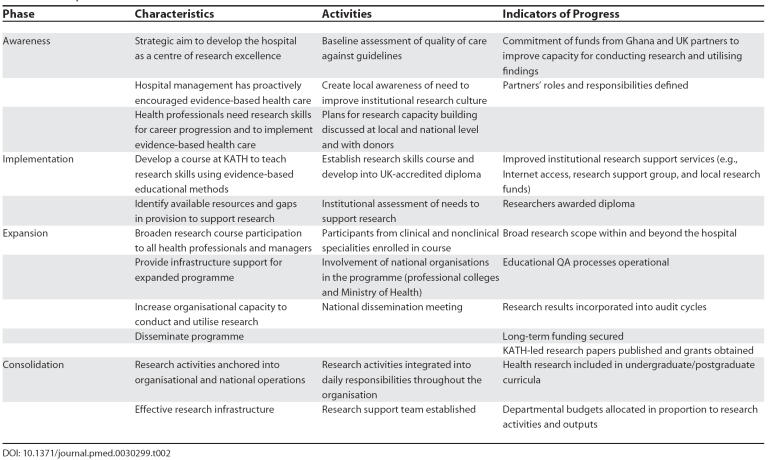
Adaptation of the Framework into a Tool That Was Relevant to the Needs of KATH

## Using the Evaluation Tool at KATH

### Identifying strengths and weaknesses in the research capacity

In the year following development of the tool, the ten health professionals undertook a research project as part of a work-based course to prepare them for the research component of their professional exams. By comparing their actual research experiences at KATH with the components itemised in the evaluation tool, they were able to identify aspects that were well supported by the institution and aspects where support was lacking or could only be provided by external facilitators. The comparison was achieved through group discussions and analysis of individual reflective statements about their research experiences, using a standard qualitative research approach known as “grounded theory”. Individual statements were scrutinised, and themes relating to research infrastructural support were extracted. Cycles of scrutinising, extracting data, and allocating it to themes were repeated until no new themes emerged [12].

A comparison between the themes that emerged from this process with the capacity-building evaluation tool identified strengths and weaknesses in the research infrastructural support. Strengths included the peer-support mechanisms within KATH, which occurred predominantly in three different contexts (peer group committees to review research proposals, small group work within course workshops, and cross-departmental research meetings). Peer support to promote work-based learning is an evidence-based educational approach [13], so the peer-support mechanisms in KATH corresponded to components of the evaluation tool. Weaknesses that emerged included gaps in knowledge concerning research resources available on the Internet, particularly systematic searching of the published literature.

### Prioritising and implementing actions for addressing gaps in the research capacity

A nominal group technique [14] was used to achieve consensus among researchers about aspects of research support that were lacking in KATH and to agree on which of these should be prioritised. For this technique, researchers used their experiences of doing research and the evaluation tool to write their own observations on areas of research infrastructure that were lacking at KATH. These were categorised into themes by the whole group and ranked according to their importance for supporting research. Gaps that were identified as priorities included provision of local statistical expertise, lack of researcher skills in critical literature reviews, and inadequate Internet access. These gaps were presented by the researchers to senior managers in KATH as a list of recommendations, and the managers incorporated activities to address these recommendations in their annual plans and budgets in 2004/2005 and 2005/2006. Progress was reviewed with the managers and the researchers during the six-month course workshops (Table 3).

**Table 3:**
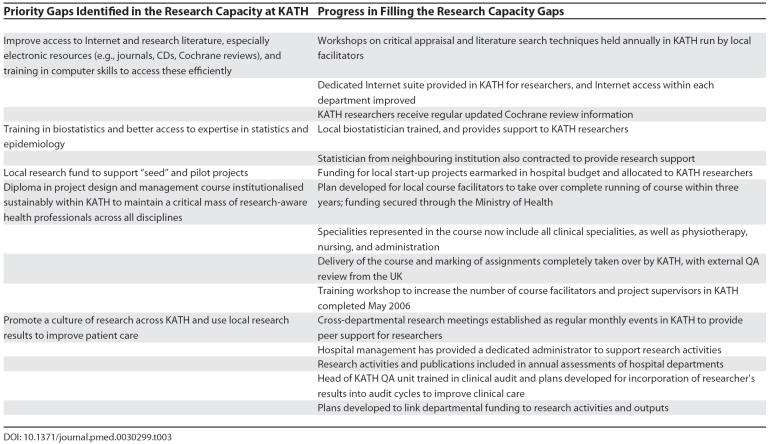
Use of the Evaluation Tool to Identify and Address Gaps in Research Capacity within 18 Months

## What Was Achieved by Using the Tool?

Progress in strengthening the research infrastructure in KATH has been achieved both for individuals and for the institution. For individuals, a course to teach research skills has been established in partnership with the Liverpool School of Tropical Medicine (LSTM). Local facilitators have been trained to run the course and funding has been secured so that within three years the course will be wholly the responsibility of KATH staff, with LSTM providing external quality reviews for the course. At an institutional level, an Internet suite has been refurbished and equipped for use by researchers, research support meetings are now a regular monthly event, and KATH has trained its own clinical biostatistician to support its researchers. Within 18 months of the original recommendations, KATH management and researchers have achieved many of the indicators of progress listed in the evaluation tool, and have developed plans to achieve the remaining indicators within the next two years. Naturally, progress some indicators, particularly those relating to using research results to improve the quality of clinical care and encouraging whole departments to be more proactive about research, will be slow and could take several years to achieve.

## Discussion

### What have we learned?

The evaluation tool has enabled researchers and hospital managers to work together to achieve a common goal of improving the research capacity in KATH. They have monitored their progress against predetermined standards and have identified and filled gaps in research infrastructure.

The evaluation tool should be flexible enough to incorporate changes in the local environment and the needs of KATH, and consequently we plan to re-evaluate and amend it within five years. Because changing the research culture of an institution is a complex process, some important components that should have been included in the tool might have been overlooked. For example, dialogue between scientists and nonscientists, as well as non-health-sector workers, is important for developing and sustaining health research capacity [3]. Such interactions are not represented in our tool, which has focused instead on building institutional capacity.

The success of the process by which this tool was developed and tested confirms the importance of the generic principles underlying effective capacity building that we extracted from the literature. We used a phased approach to engage stakeholders in identifying strengths and weaknesses, and then to develop, implement, and monitor action plans to address these gaps [6]. Part of this process involved identifying and strengthening existing processes and building up local resources, rather than developing new parallel systems [15]. This strengthening process included formalising the peer-support meetings that researchers had found so helpful, and expanding the existing Internet facilities. The process is a good example of a genuine partnership for problem solving that is built on trust, common interest, long-term commitment, and shared responsibilities and obligations [16,17]. Although funding for the process was initially shared between KATH and LSTM, KATH has maintained ownership and leadership, and is now totally funding the capacity-building process. Each partner had clearly delineated roles, and mechanisms and timescales for transfer of skills from LSTM to KATH staff were agreed on early in the process.

Two important criteria for this project's success were the motivation of the researchers and the strong leadership and commitment of KATH managers. Participation of all stakeholders in the design of evaluation indicators is recognised to promote motivation and commitment (see Chapter 7 [18]). The rate of progress is likely to slow down over the next few years, as the institutional shift towards research begins to involve individuals who might not have the high motivation of the managers and researchers, but the tool nevertheless provides a means for maintaining focus on achieving some of the more difficult indicators.

### How transferable are these lessons and the tool?

The generic principles of effective capacity building—phased approach, strengthening existing systems and partnerships for problem solving—were derived from contexts that were not health sector–specific, and yet they have been applied successfully here. However, the evaluation tool was developed for the context of health research at KATH, and its value and transferability in other contexts would need to be assessed.

Although the framework from which the tool was derived incorporated all the elements of a research process, such as problem identification, priority setting, and research use (see page 16 in [2]), the specific components used to produce the operational tool would need to be adapted to suit the specific needs of other institutions. Monitoring and evaluation is the most difficult and neglected component of capacity-building programmes because they can take over 20 years to achieve their objectives [8], and some outcomes, such as organisational culture, are difficult to measure [19]. Different users of evaluations will have different priorities, and the use of an evaluation tool helps to promote agreement on the purpose of the evaluation and the indicators [20]. The major advantages of our tool are that it enables an institution in a developing country to set its own priorities, to have control over local capacity building [21], and to evaluate progress in building capacity from its own perspective rather than from that of an external agency.

## Supporting Information

Dataset S1 Further Details of the Literature Review on Capacity Building (52 KB DOC).

## Abbreviations

EBM: evidence-based medicine
KATH: Komfo Anokye Teaching Hospital
LSTM: Liverpool School of Tropical Medicine
QA: quality assurance
WHO: World Health Organization
