# Supplementary material for: Evaluating Health Research Capacity Building: An Evidence-Based Tool
Source: PLoS Med. 2006 Jul 18;3(8):e299. doi: 10.1371/journal.pmed.0030299 (PMC1502158; doi:10.1371/journal.pmed.0030299)
Supplement: Supplementary file 1 [file pmed.0030299.sd001.doc]

# Data Supplement 1

# Draft of capacity building literature review

# Section 1. Review of current knowledge about capacity building in general

Many definitions have been applied to capacity building but they vary depending on whether the programmes have focussed on the ‘micro’ (e.g. an individual NGO), meso (e.g. institution or organisation) or ‘macro’ level (e.g. nation building strategies).^2^ One of the most widely used recent definitions is *‘an ability of individuals, organisations or systems to perform appropriate functions effectively, efficiently and sustainably’*.^3^ Strategies for implementing capacity building therefore need to focus on the questions - capacity for what?, for who? and how? There is almost no evidence-based information about how to implement or evaluate this holistic type of capacity building. Much of the literature is based on retrospective reports of external consultants. There is virtually no information specifically about building research capacity for health and the developing country perspective is almost never represented.

# General principles about best practice in capacity building programmes

# *Phased approach*

Similar to the principles of introducing change management programmes, capacity building should comprise three broad, overlapping components^3^:

- Involvement of all stakeholders in an assessment of capacity gaps and available resources
- development of strategies and actions to fill these gaps that build on existing resources
- evaluations that encompass indicators of outputs, processes and outcomes

Strengthening existing processes

One of the most consistent and important messages from the literature is that capacity building is not just about training groups or individuals. Because of the complexity of ensuring inclusion of all levels involved in the capacity building process, programmes are more likely to be successful if they start with the main focus, and then incrementally incorporate expanding circles of individuals, institutions and systems.^4^ To be able to adjust to new environments and challenges, capacity building must be a continuous iterative and flexible process, focussed on enhancing local ability to solve problems and to define and achieve development needs.

# *Partnerships for problem solving*

Until recently, rhetoric around ‘partnership’ for capacity building generally meant wealthy organisations retaining control and leadership while donating funds and expertise to poorer ‘beneficiary’ partners. Genuine partnerships are built on trust, common interest, long-term commitment and shared responsibilities and obligations.^5^ There is evidence that capacity building cannot be effective or sustained unless both partners have similar concepts^6^ and local partners take ownership and leadership.^3,7^ There are many examples of collaborations providing added value but because capacity building focuses on internal processes, external agencies can only have a catalytic or lubricating role; they cannot steer the process. The role of external expertise in capacity building is therefore to facilitate development of local skills through learning by doing.

# Evaluating effectiveness of capacity building programmes

Monitoring and evaluation is the most neglected component of capacity building programmes. Literature is scarce and experience very limited. There are major conceptual and methodological difficulties in designing indicators to evaluate the effectiveness of such complex programmes. As strategies to build capacity become more sophisticated, it is increasingly difficult to adapt and apply existing evaluation methodologies.^8^ Available information tends to reflect the reporting requirements of donor agencies rather than evaluating the practical impact of programmes from the Southern partners’ perspectives.

#### Evaluation strategies

Because each research capacity building programme is focussed around a specific task and is associated with unique constraints it is not possible or desirable to develop a generic model. This means that most capacity building is experimental and therefore it should be rigorously evaluated. Capacity building programmes include easily measurable outputs, such as training, as well as less tangible outcomes such as organisational culture. Assessments of effectiveness therefore need to be imaginative, innovative and to evaluate not just competence, but expertise. Capacity building results can take over 20 years to come to fruition^4^ so it is essential that evaluations achieve an appropriate balance of input, process (i.e. measuring how individuals and organisations behave), output and intermediate outcome indicators.

#### *Local participation to ensure effective evaluation*

Different users of evaluations will have different priorities so it is important to agree on the purpose of the evaluation and to develop agreed indicators.^9^ One of the major obstacles to achieving consensus on evaluating capacity building is a lack of understanding about the relationship between capacity and performance,^10^ and particularly the need to reflect the African perspective. Carefully designed participatory evaluations could be used as tools to increase motivation and commitment from local partners.^11^

## **Assessing donor effectiveness**

Donors need to take a pragmatic, iterative approach and adopt very flexible administrative systems to cope with the challenges of implementing and holistically evaluating capacity building programmes. A detailed self-assessment tool has been developed that enables donors to monitor their progress in implementing their capacity development principles.^12^

# Section 2. Review of current knowledge about building capacity for health research

‘Only 10% of the global health research money is spent in the South, which shoulders 90% of the global burden of disease’.^13^ We are still waiting for the ‘quantum leap in capacity building’ identified in 1998 by the Director General of the WHO as a priority issue for the international community.^14^ Almost all the reports about building capacity for research are limited to micro-level operations (i.e. training to enhance research skills).

**Building research capacity …..**

***‘For what’?***

The goal of research capacity building is to ‘promote evidence-based decision making at all levels of the health field’.^14^ Using this as the basis to adapt the generic definition, building capacity for health research can be defined as *‘an ability of individuals, organisations or systems to perform and utilise health research effectively, efficiently and sustainably’*.

***‘For who?’***

For health research to have an impact, research outputs need to be put into practice on a wide scale. Consequently, improving the accessibility and use of research should receive as much, if not more, attention as strengthening the performance of research.^14^ For every scientist trained, many hundreds of individuals (including health professionals, policy makers and funders) need to be educated about how to use research results to make evidence based decisions.

The research community recognises that international networking between scientists is important.^16^ However, experience suggests that collaborations between scientists alone are unlikely to achieve sustainable and effective health research capacity building unless they are embedded within existing local systems. This requires dialogue between scientists and non-research, and even non-health, sectors. Important mechanisms for developing and sustaining research capacity^14^ include training of individuals, adequate career structures and remuneration, research support infrastructure, partnerships and networking and involvement by policy makers. Health research capacity building ‘for who’? should therefore include:

- individual researchers who need to advance their careers
- institutions wanting to improve quality of patient care
- other sectors and organisations that interact with health institutions
- policy makers needing to plan and budget on the basis of effective, evidence-based strategies

.

***And ‘how?’***

One of the most difficult challenges in health research capacity building is to utilise existing capacity and systems without generating confrontations and avoiding diverting scarce resources away from other critical areas. This dilemma is exemplified by the dilemmas generated by the scaling up of anti-retroviral therapy in countries already unable to provide a basic level of routine heath care.^9^

In health research, capacity building has tended to concentrate on the straightforward approach of external training of scientists, without concomitant strengthening of local training systems and without including organisations and individuals that need to pick up, understand and act on research results. Experiences from health promotion practice suggest that sophisticated, innovative and flexible strategies are required to facilitate learning for all these players, including experiential experience in the workplace. Mechanisms that can be used to encourage use of research results include accessible research support, efficient research dissemination and research secondments.^15^

Research training in developing African countries is often not an integral part of professional training. Education in research awareness, audit and evaluation therefore needs to be introduced into all under-graduate and post-graduate health curricula as part of building research capacity. Health managers need to foster institutional and national cultures that are supportive of research and to regard it as integral to the planning and delivery of high quality health care. Institutionalisation of health quality assurance programmes has demonstrated that factors such as policy, leadership, core values and resources, contribute to an enabling environment^17^ and can be applied at all levels.

# *Partnerships for building health research capacity*

Conventional methods of health research capacity building have often been restricted to one-off, externally driven training and technical assistance. This has created a tension, particularly for donors, between enhancing local ownership and using external experts to deliver outputs. Genuine partnerships are more complex and time consuming than contracting a partner to execute specific tasks.^6^ It is rarely acknowledged that African research capacity exists and that North-South partnerships can build each others’ capacity.^18,19^ Key contributions from Southern collaborators to research partnerships include initiating the research agenda and adapting the research process to fit local systems and cultures. One area for building research capacity that has been long recognised but particularly overlooked is the lack of first author publications by Southern, and particularly African, researchers.^20,21,22^

**Measuring effectiveness of research capacity building programmes**

The scanty literature on evaluation of health research capacity building emphasises micro level achievements such as the choice of research trainees.^14^ The holistic and flexible nature of sustainable research capacity building programmes mean that they require much broader innovative and appropriate evaluation mechanisms, which incorporate meso and macro levels and which draw heavily on qualitative research methodology and educational theory. There is published evidence, predominantly from education and management disciplines, describing best practice models for evaluating some of the components of capacity building, but there is little evidence of their application to health research capacity building programmes. For this to be possible, the constituent parts of these highly complex programmes will need to be teased out so that appropriate best practice qualitative and quantitative methodologies can be adapted and applied to the health research context. (figure 1)

## **Section 3. Developing a framework for research capacity building programmes**

Specifications for the framework

- needs to guide planning, implementation and evaluation of health research capacity building programmes.
- The framework should incorporate available evidence about building health research capacity.
- Adapt from frameworks used by quality assurance^17^ and management disciplines.^23^
- Similar to the model of developing individual expertise, which progresses through overlapping phases of ‘competency’ (knows how), ‘proficiency’ (shows how), ‘global development’ (does) and ‘expertise’ (does well).^24^
- Also incorporate indicators of effectiveness (figure 1)

Figure 1. Potential indicators for evaluating health research capacity building programmes

|  | Indicators | |
| --- | --- | --- |
| **Level for evaluation** | **Process/outputs** | **Outcome** |
| Micro (individuals) | - Publications - Grant income | Research leaders with ability to conduct independent research |
| Meso (organisations) | - Local training programmes (how to evaluate and use research as well as how to do it) - Availability of research resources - Efficient access to electronic and other resources - Institutional research ‘culture’ (e.g. committed budget, ethics committee, study leave policies, research orientated CPD programmes) - Research awareness included in undergraduate curricula - Strong researcher-practitioner links | Locally generated sustainable, effective, independent research  Evidence-based decision making to improve clinical outcomes |
| Macro (national) | - Effective communication channels between researchers and policy makers - Evidence based policies and practices - Career structure and remuneration for scientists - Research policy group | Evidence-based decision making to improve clinical outcomes and health indicators |
